# Genetic Diversity of Chinese Giant Salamanders Under the Context of Translocation Using Novel Development of Genomic SSR Markers

**DOI:** 10.1002/ece3.71375

**Published:** 2025-05-08

**Authors:** Mingyao Zhang, Yixing Xie, Qiang Zhou, Cheng Wang, Zhiyong Deng, Fengjiao Wu, Zhiqiang Liang, Ying Wei, Wansheng Jiang

**Affiliations:** ^1^ Hunan Engineering Laboratory for Chinese Giant Salamander’s Resource Protection and Comprehensive Utilization, College of Biology and Environmental Sciences Jishou University Jishou China; ^2^ National and Local United Engineering Laboratory of Integrative Utilization Technology of Eucommia Ulmoides Jishou University Zhangjiajie China; ^3^ Hunan Zhangjiajie Giant Salamander National Nature Reserve Affairs Center Zhangjiajie China; ^4^ Vertebrate Zoology Laboratory Hunan Normal University Changsha China; ^5^ Zhangjiajie National Forest Park Zhangjiajie China; ^6^ Fisheries Research Institute of Hunan Province Changsha China; ^7^ College of Wildlife and Protected Area Northeast Forestry University Harbin China

**Keywords:** *Andrias* spp., draft genome, genetic diversity, microsatellite, reintroduction

## Abstract

Genetic diversity is crucial for assessing biodiversity and understanding the evolutionary potential of threatened species like the Chinese giant salamanders (*Andrias* spp., CGS), which are among the most endangered amphibians globally. With extensive translocation efforts aimed at conservation, it is essential to assess genetic diversity using molecular markers to gauge potential impacts on the original populations. In this study, 15,140,972 genomic scaffold sequences of CGS were assembled using next‐generation sequencing, revealing 316,313 simple sequence repeat (SSR) loci, predominantly dinucleotide repeats. From 200 randomly synthesized SSR primer pairs, 19 markers with moderate to high polymorphisms were validated and selected to evaluate the genetic diversity of CGS based on 60 wild individuals from six sampling sites in the Hunan Zhangjiajie Giant Salamander National Nature Reserve. Results highlighted the highest diversity in the Jinbianxi population, a long‐term reinforcement site, and the lowest in the Wumuyu population, a historically natural site. Genetic differentiation was most pronounced between the populations of Wumuyu and another historically natural site, Yuanzi, contrasting with lower differentiation between two recently reintroduced sites, Xixiping and Xiangshi. The genetic structure of the Jinbianxi population suggests potential hybridization events between distinct genetic lineages or species, along with long‐term translocation practices. This study introduces a large set of genomic SSR markers of CGS, highlighting the significance of reliable markers for evaluating its genetic dynamics. It also stresses the necessity of continuous monitoring, assessment, and management of genetic diversity to enhance conservation strategies effectively.

## Graphical abstract

This study developed 19 novel genomic SSR markers for the critically endangered Chinese giant salamander (*Andrias* spp.) using next‐generation sequencing, revealing 316,313 SSR loci. Analysis of 60 wild individuals across six sites in Hunan Zhangjiajie Giant Salamander National Nature Reserve identified the highest genetic diversity in the long‐term translocated Jinbianxi population and the lowest in the natural Wumuyu site, with significant differentiation between natural populations compared to recently reintroduced groups. The findings emphasize the need for genetic monitoring to mitigate hybridization risks and improve conservation strategies for this imperiled species.

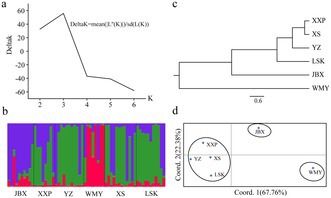

## 1 Introduction

Chinese giant salamanders (*Andrias* spp., CGS), which can reach a maximum length of 2 m and weigh up to 50 kg, are the world's largest extant amphibians (Jiang et al. 2022). Taxonomically, they belong to the Cryptobranchidae family in the order Caudata, often regarded as the earliest crown group of salamanders and referred to as living fossils (Dong et al. 2011; Gao and Shubin 2003). CGS were once widespread in central and southern China, inhabiting an area roughly between 23.5°‐35° N and 100°‐120° E (Murphy et al. 2000), including the three major river basins: the Yellow, Yangtze, and Pearl rivers (Jiang et al. 2023). Since the 1950s, habitat degradation, fragmentation, and overharvesting have led to a significant decline in both the distribution range and population numbers of wild CGS. Many populations are now on the brink of extinction, with some already locally extirpated (Wang et al. 2004). Wild CGS populations have been classified as Class II state‐protected animals since 1988 and are also included in the Chinese Red Book of Amphibians and Reptiles (Jiang et al. 2016). They have been listed as critically endangered (CR) on the IUCN Red List of Threatened Species since 2004, with their status reaffirmed in the most recent assessment (IUCN SSC Amphibian Specialist Group 2023).

For a considerable period, CGS was believed to comprise only one species, namely, *Andrias davidianus* . However, recent genetic studies have revealed the presence of five to seven distinct cryptic species (Yan et al. 2018) or divergent phylogenetic lineages (Liang et al. 2019) within the *A. davidianus* species complex. Building upon these phylogenetic frameworks, several subsequent studies have revised the taxonomic classification of CGS. Three newly recognized species have been distinguished from the original *A. davidianus* : *Andrias sligoi* (Turvey et al. 2019), *Andrias jiangxiensis* (Chai et al. 2022), and *Andrias cheni* (Gong et al. 2023). However, unlike many traditional morphological taxonomy methods, the taxonomic revisions of CGS mentioned above were primarily, if not exclusively, based on molecular analyses (Turvey et al. 2019). The morphological diagnoses provided for these species were also limited to holotypes due to potential morphological ambiguity arising from translocated animals or hybrids (Chai et al. 2022; Gong et al. 2023).

Genetic diversity analysis has been a prominent aspect of CGS research history, as evidenced by the variety of molecular markers employed over time. These include isoenzymes, mitochondrial DNA, random amplified polymorphic DNA (RAPD), amplified fragment length polymorphism (AFLP), simple sequence repeats (SSRs), mitochondrial genome sequencing, and single nucleotide polymorphisms (SNPs). For instance, Murphy et al. (2000) utilized isoenzyme electrophoresis and mitochondrial DNA sequencing to investigate genetic differences among six CGS populations in China, identifying the Huangshan population as distinct in terms of genetic distance and phylogeny. Lin et al. (2003) conducted genetic analyses of both wild CGS populations and artificially bred offspring using RAPD, while Yang et al. (2011) assessed the genetic differentiation of wild CGS populations across five provinces using AFLP techniques. SSRs have also been utilized in numerous studies to perform genetic analyses of CGS (Meng et al. 2012; Wang et al. 2014). Most other studies, however, have primarily relied on mitochondrial gene fragments. It has often revealed relatively low genetic diversity in CGS, with some geographical clades showing distinct differentiation (Fang et al. 2008; Huang et al. 2012; Tao et al. 2005; Tao et al. 2006; Wu et al. 2017). Nevertheless, these studies have typically been based on limited samples from few sites and have utilized a narrow range of informative molecular markers. More comprehensive understandings of the genetic differentiation of CGS in China emerged only when studies incorporated larger sample sizes covering the entire distribution range and employed additional molecular markers such as SNPs (Yan et al. 2018) and complete mitochondrial genomes (Liang et al. 2019), in combination with traditional mitochondrial gene fragments. This approach led to the emergence of the seven phylogenetic lineage (or species) hypothesis, which subsequently formed the basis for taxonomic revision studies of CGS (Chai et al. 2022; Gong et al. 2023; Turvey et al. 2019).

In efforts to protect and restore wild populations of CGS, the practice of translocating or reintroducing farm‐bred individuals into the wild has been carried out in many provinces of China (Shu et al. 2021). While the primary objective of CGS translocation is conservation, and it has achieved significant success in some areas (Liu et al. 2021), there is a risk that it may accelerate the extinction of local phylogenetic lineages or species through genetic homogenization if the genetic diversity of farm‐bred individuals is limited (Yan et al. 2018). Conversely, reintroductions involving mixed sources can enhance genetic diversity but may also result in a proliferation of hybrid offspring or compromise the genetic integrity of original populations (Shu et al. 2021). Therefore, the practice of translocation requires careful consideration of both the potential conservation benefits and the various impacts of human‐mediated genetic changes (Zhou et al. 2024). In particular, given the ongoing changes in the taxonomic system of CGS, there is an urgent need for timely assessments of genetic diversity in the context of widespread translocation efforts.

In a broad sense, genetic diversity serves as an indicator of a species' evolutionary potential. High levels of genetic diversity enhance a species' capacity for evolutionary adaptation, whereas low levels render them less resilient to environmental changes (Binks et al. 2015). Assessing genetic diversity can also provide valuable insights for the conservation of threatened species, including managing genetic sources for both pre‐ and postrelease individuals of CGS (Cunningham et al. 2016). Typically, mitochondrial gene sequences have been used as molecular markers to reconstruct phylogenetic lineages and evaluate the genetic diversity of CGS. However, since mitochondrial DNA is exclusively found in mitochondria and strictly follows maternal inheritance (Garone et al. 2018), it may not offer a comprehensive representation of the overall genetic information of CGS. Additionally, in situations where rapid genetic introgression occurs (Yan et al. 2018) or when risks such as outbreeding depression arise from mixed‐source translocations of CGS (Shu et al. 2021), mitochondrial genes alone may not adequately reflect the dynamic trends of genetic changes.

SSRs, also known as microsatellites or short tandem repeats (STRs), are composed of two to six base pair motifs that are tandemly repeated several times in the genome. They are generally abundant, highly polymorphic, codominant, and repeatable, and provide proper genome coverage, endowing them with certain advantages and broad applications (Jiang et al. 2014; Tóth et al. 2000). However, studies on SSR molecular markers in CGS have been relatively scarce compared to those using other markers, such as mitochondrial gene sequences. This is mainly because of the limited availability of practical SSR markers. Previously, the limited SSR markers utilized in CGS were primarily developed, if not exclusively, through a method known as Fast Isolation by AFLP of Sequences Containing repeats (FIASCO) (Meng et al. 2012; Wang et al. 2014). However, developing SSRs using this approach was typically expensive, time‐consuming, and labor‐intensive due to the construction of enriched libraries and the use of cloning and Sanger sequencing (Jiang et al. 2018). In comparison, isolating microsatellite loci using next‐generation sequencing (NGS) technologies provides an ideal alternative, as it can generate a large amount of sequences efficiently and at low cost (McCombie et al. 2019). With the rapid development of NGS, the successful identification of SSR markers based on the genome has been carried out in various animals, including birds (Huang et al. 2016), amphibians (Chen et al. 2018a), fish (Huang et al. 2019) and more. However, the selection of effective SSR markers for genetic diversity studies remains challenging, as these markers must demonstrate sufficient polymorphism at the population level to ensure statistical robustness. To advance conservative genetic studies of CGS in the future, particularly in the context of translocation, SSR markers specific to CGS were identified for the first time based on the draft genome of CGS. The present study aimed to: (1) sequence and assemble the draft genome of CGS using NGS; (2) identify and screen SSR markers based on draft genome data; (3) analyze and assess the genetic diversity of several populations of CGS using the polymorphic SSR markers developed in this study.

## 2 Materials and Methods

### 2.1 Sample Collections and Sampling Sites

**FIGURE 1:**
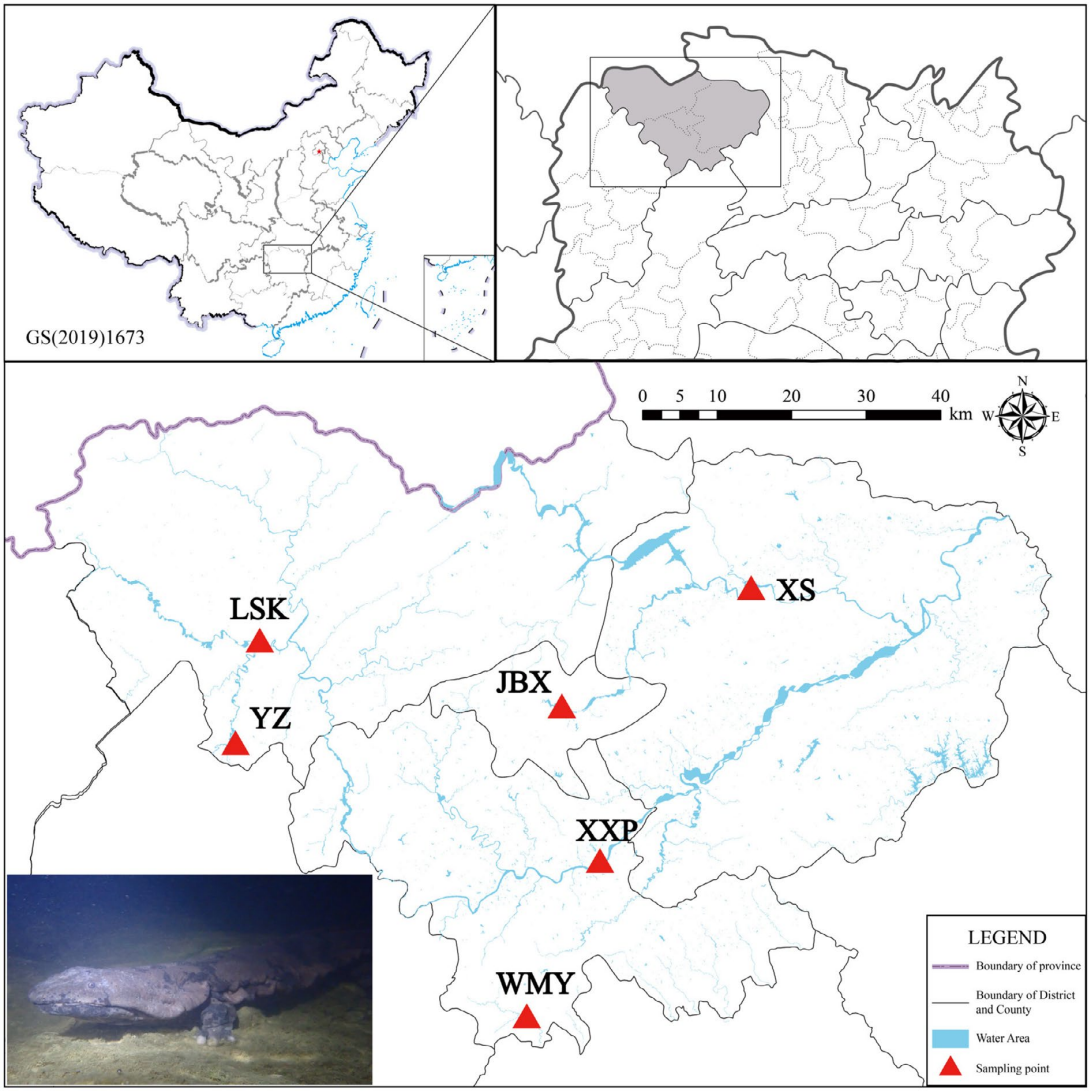
The six sampling sites located in Zhangjiajie City of Hunan Province. *Note:* The inset in the lower‐left corner shows an individual of CGS found at site JBX.

The samples used in this study were collected within the Hunan Zhangjiajie Giant Salamander National Nature Reserve during surveying and monitoring periods spanning from 2020 to 2023. Specimens were temporarily held for tissue sample collection and then released on‐site. Generally, tissue samples were obtained through skin swabbing, following an optimized nondisruptive DNA sampling procedure developed in our previous study (Wang et al. 2022). In the case of specimens with large body sizes, a small strip of caudal fin was clipped from several individuals. Additionally, muscle samples were extracted from a farm‐bred individual (voucher no. JWS2021322) for genomic sequencing. A total of 60 wild samples were collected from six distinct sampling sites within the Hunan Zhangjiajie Giant Salamander National Nature Reserve (Figure 1). These sampling sites could be categorized into three types based on the impact of previous translocation efforts: (1) historically natural sites, comprising Wumuyu (WMY, *n* = 8) and Yuanzi (YZ, *n* = 11), both of which harbor populations within natural caves and have seen minimal involvement in translocation practices; (2) long‐term reinforcement sites, exemplified by Jinbianxi (JBX, *n* = 10), which was historically a natural site but has been subjected to translocation efforts for several years, potentially resulting in the majority of current individuals originating from translocations; and (3) recently reintroduced sites, represented by Xixiping (XXP, *n* = 8), Xiangshi (XS, *n* = 12), and Liangshuikou (LSK, *n* = 11), where translocation has only recently been initiated with the aim of population recovery.

### 2.2 DNA Extraction, Sequencing, and Assembly

All samples were preserved in 100% alcohol at −20°C until DNA extraction. Total DNA extraction was performed using the DNeasy Blood & Tissue Kit (Qiagen, Hilden, Germany), and the integrity and purity of the DNA were assessed through agarose gel (1%) electrophoresis and spectrophotometry (NanoDrop 2000, Thermo Fisher Scientific, USA), respectively. Genome sequencing was conducted in paired‐end mode on the DNBSEQ‐T7 platform by a commercial biotechnology service provider, aiming to yield approximately 400 Gb of raw reads with a read length of 150 bp. Adapter sequences for sequencing were removed, and reads containing low‐quality bases were filtered out using the fastp software (Chen et al. 2018b). The quality of the clean reads was evaluated using FastQC (https://www.bioinformatics.babraham.ac.uk/projects/fastqc/). Subsequently, the clean reads were assembled into contigs and then converted into scaffolds using a four‐step assembly strategy in SOAPdenovo2 (Luo et al. 2012). Assembly statistics, such as contig N50 and scaffold N50, were acquired using QUAST (Mikheenko et al. 2018).

### 2.3 SSR Markers Development and Validation

The dataset of assembled scaffold sequences was used to screen SSR loci using the Perl script software MISA (Thiel et al. 2003). SSR loci were sought for dinucleotide to hexanucleotide repeat motifs with a minimum repeat number of 6, 5, 5, 5, and 5, respectively. For compound SSR loci, the interval between two repeat motifs was set to less than 100 bp. Primer pairs were designed using the Primer3 software (Untergasser et al. 2012), following specific criteria: primer length was constrained between 18 and 23 bp with 20 bp as the optimum, melting temperature ranged from 50°C to 62°C, GC content was between 40% and 60%, and product size was between 100 and 280 bp.

A total of 200 primer pairs were randomly selected for further validation. Initially, all primer pairs were tested via PCR evaluation using two individuals known to be from different phylogenetic lineages. Primer pairs that produced the expected product length with a bright, single band were selected for further analysis. The PCR amplifications were conducted in a 25 μL reaction system, comprising 1 μL of DNA template, 12.5 μL of PCR mix, 1 μL of each primer pair, and 9.5 μL of ddH_2_O. The PCR conditions were as follows: initial denaturation at 95°C for 5 min, followed by 35 cycles of denaturation at 94°C for 30 s, annealing at primer‐specific Tm for 30 s, and extension at 72°C for 30 s, with a final extension at 72°C for 5 min. Subsequently, primer pairs that produced accurate and distinct electrophoretic bands were chosen for polymorphism evaluation. This evaluation was conducted on 10 individuals randomly selected from various populations. The 5′ end of the forward primer of these primers was labeled with FAM. PCR products were analyzed on an ABI3730XL Genetic Analyzer sequencer. Allele assignment and fragment analysis were conducted using GeneMapper software version 4.1 (Applied Biosystems). Finally, primer pairs that generated polymorphic alleles with appropriate diversity were further filtered and retained, while SSR loci lacking polymorphism among all the tested samples were discarded.

### 2.4 Genetic Diversity Analysis

Genetic diversity was assessed based on the polymorphic primer pairs selected through the SSR validation procedures described above, using 60 individuals of wild CGS from six sites. Genetic variation, including the number of alleles (Na), number of effective alleles (Ne), observed heterozygosity (Ho), expected heterozygosity (He), Shannon's information index (I), and Nei's genetic diversity and genetic distance, was calculated using Popgene 1.32 (Yeh and Boyle 1997). Polymorphism information content (PIC) was computed using the Cervus software (Kalinowski et al. 2007). To examine the genetic structure of CGS, we employed a Bayesian Markov Chain Monte Carlo approach to estimate the number of genetic clusters. This model‐based analysis was conducted in STRUCTURE v.2.3 (Hubisz et al. 2009). For each K value ranging from 2 to 6, we ran 10 replicates with a burn‐in length of 50,000 iterations, followed by 100,000 iterations for each chain. We employed the admixture model and assumed correlated allele frequencies between groups. Furthermore, the genetic diversity and population structure of the CGS accessions were investigated through principal coordinate analysis (PCoA) using GenAlEx 6.5 (Peakall and Smouse 2012).

## 3 Results

### 3.1 Genome Sequencing and Assembly

Approximately 410 Gb of raw data was generated through an NGS approach. Evaluation of the raw data quality revealed normal values for GC content (48.69%), as well as bases above Q20 (96.53%) and Q30 (89.60%), indicating suitability for further processing. Following the removal of sequencing adapters and low‐quality raw reads, approximately 99.9% of the cleaned paired reads were retained. Utilizing approximately eightfold coverage of the CGS genome, a *de novo* assembly was conducted, resulting in a shallow draft genome that was nonetheless sufficient for SSR development. The assembly process generated 15,680,081 contigs and 15,140,972 scaffolds using a four‐step assembly strategy with SOAPdenovo2. The largest contig and scaffold lengths were 8280 and 21,326 bp, respectively. Within the final dataset of scaffold sequences, the total assembly length was 2,894,485,933 bp. The scaffold N50 and N75 lengths were 744 and 572 bp, respectively, and the scaffold L50 and L75 were 133,181 and 256,930, respectively (Table 1).

**TABLE 1:** Summary statistics of the assembled shallow draft genome of CGS.

No.	Items	Values
1	Total raw reads	2.728 G
2	Total number of bases (bp)	409.267 G
3	Clean reads proportion (%)	99.928%
4	Total number of scaffolds	15,140,972
5	Assembly length (bp)	2,894,485,933
6	Largest scaffold size (bp)	21,326
7	L50 (bp)	133,181
8	L75 (bp)	256,930
9	N50 (bp)	744
10	N75 (bp)	572
11	GC content (%)	48.51%
12	Q20%	96.501%
13	Q30%	89.538%

### 3.2 Development and Characterization of SSRs

**FIGURE 2:**
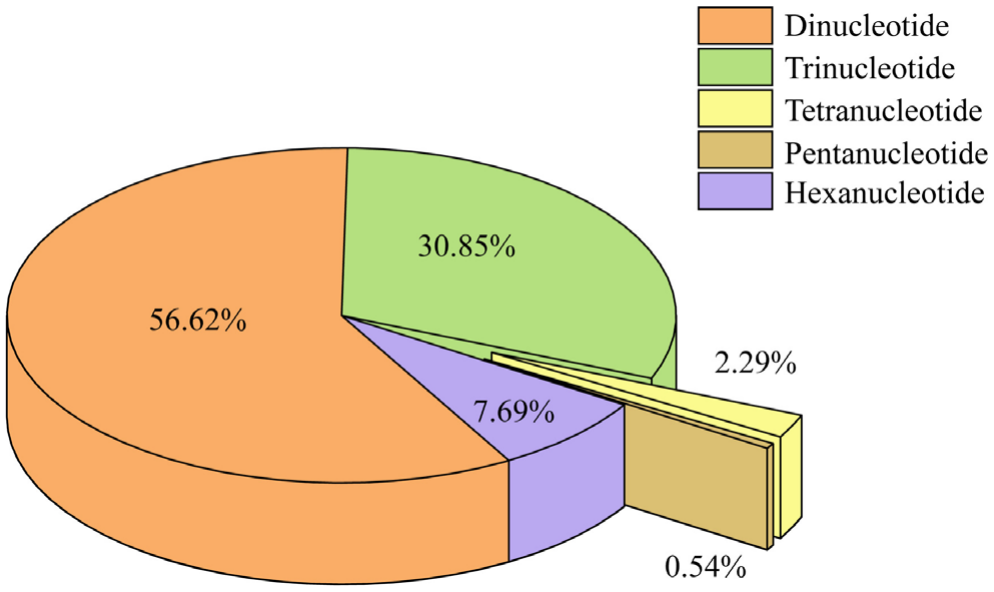
Frequencies of different SSR motif types.

A total of 316,313 SSRs were identified using the MISA software after removing redundancy (Table S1). These SSRs comprised 185,438 dinucleotide repeats (Di‐SSRs, 56.62%), 97,577 trinucleotide repeats (Tri‐SSRs, 30.85%), 24,327 hexanucleotide repeats (Hexa‐SSRs, 7.69%), 7248 tetranucleotide repeats (Tetra‐SSRs, 2.29%), and 1723 pentanucleotide repeats (Penta‐SSRs, 0.54%) (Figure 2). Among them, 15,041 repeats were identified as compound SSR loci. The total length of all SSRs was 6,057,876 bp, accounting for 0.21% of the total assembly scaffold length. The relative abundance and relative density of SSRs were 109.28 loci/Mb and 2093.90 bp/Mb, respectively. The average distance between each pair of SSRs within the same scaffold was 2.35 Kb.

**FIGURE 3:**
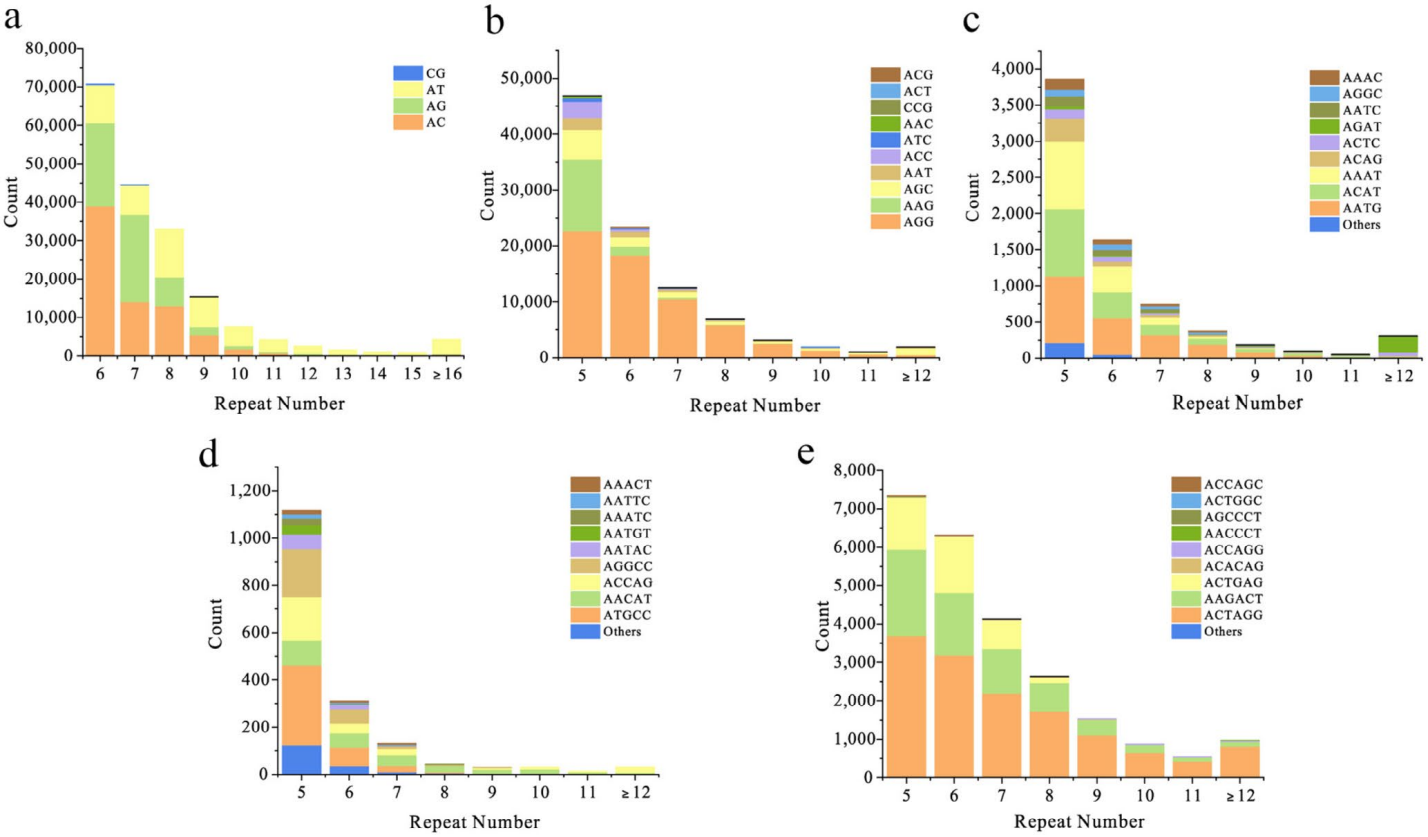
Distributions of repeat units for different SSR types in the draft genome of CGS. *Note:* a to e represent the distributions of repeat units in Di‐SSRs, Tri‐SSRs, Hexa‐SSRs, Tetra‐SSRs, and Penta‐SSRs, respectively. The X‐axis represents the number of repeat units, and the Y‐axis represents the count of that SSR loci. The repeat bases of each type are presented in different colors.

Further analysis of the proportions of each type of SSR (considering sequence complementary) in CGS revealed significant differences in both the types of bases and repeats. Among Di‐SSRs, the base type AC emerged as the dominant category, with the highest number (750,593), accounting for 40.76% of the total Di‐SSRs. The AG and AT categories followed closely behind, representing 30.04% and 29.13% of the total Di‐SSRs, respectively. Conversely, the CG category was the least abundant, with only 125 occurrences, accounting for a mere 0.07% of Di‐SSRs. Among Tri‐SSRs, the AGG category exhibited the highest number (62,398), constituting over half (63.95%) of the Tri‐SSRs, while other Tri‐SSRs did not show significant proportions. Within Tetra‐SSRs, AATG, ACAT, and AAAT were the top three categories, collectively accounting for 70% of the total numbers. Regarding Penta‐SSRs and Hexa‐SSRs, ATGCC and ACTAGG were the most abundant categories, representing 26.18% and 56.76% of the corresponding types, respectively (Table 2). Among all types of repeats, the 10 most abundant SSR repeats occurred in the following sequence: AC, AGG, AG, AT, AAG, ACTAGG, AGC, AAGACT, AAT, and ACTGAG, representing the most common SSRs in the CGS genome. Additionally, across all five types of repeats, the number of repeat units exhibited a similar pattern, with SSR loci decreasing correspondingly as the repeat units increased (Figure 3).

**TABLE 2:** The three most frequent repeat categories in each SSR type.

SSR Types	Category	Quantity	Proportion
Dinucleotide repeats	AC	75,593	40.76%
	AG	55,705	30.04%
	AT	54,015	29.13%
Trinucleotide repeats	AGG	62,398	63.95%
	AAG	15,041	15.41%
	AGC	10,930	11.20%
Tetranucleotide repeats	AATG	2040	28.15%
	ACAT	1649	22.75%
	AAAT	1422	19.62%
Pentanucleotide repeats	ATGCC	451	26.18%
	AACAT	294	17.06%
	ACCAG	288	16.72%
Hexanucleotide repeats	ACTAGG	13,809	56.76%
	AAGACT	6636	27.28%
	ACTGAG	3715	15.27%

### 3.3 SSR Makers Selection and Validation

Following a preliminary regular PCR evaluation, 116 of the 200 randomly selected primer pairs successfully amplified bands, as confirmed by 1% agarose gel electrophoresis. Subsequently, 71 primer pairs consistently amplified a specific single band in the two tested individuals. Through further polymorphism evaluation, 26 of the 71 primer pairs produced polymorphic alleles, while the remaining 45 primer pairs exhibited monomorphism in the 10 tested individuals. Among the 26 primer pairs that produced polymorphic alleles, 7 showed a low level of polymorphism (PIC < 0.25). Consequently, all monomorphic and low polymorphic loci were discarded due to their insufficient suitability as SSR molecular markers in the following analysis. Ultimately, a total of 19 SSR primers showing significant polymorphism (PIC > 0.25) were chosen and kept for assessing the genetic diversity of CGS (Table 3).

**TABLE 3:** Information of 19 polymorphic SSR loci in CGS.

Locus	Repeat motif	Primer sequence (5′–3′)	Tm (°C)	PIC
Y15	(AT)6	F: AAAAACCTTTGGTTGTTGTCTAGA	53	0.441
		R: GTGAGATAAGGTTGCATTGCAA		
Y54	(CA)31	F: CCACACACACACACACACAC	55	0.341
		R: CGTGCAGTGCTTTGCTACAG		
Y58	(CTG)33	F: TTTAGCCTGCTTCTGCTGCT	53	0.375
		R: CGGAAGGATCGTGCAGCA		
Y62	(GCA)28	F: GCCAGGCCAAAACACAAAGT	54	0.375
		R: CCTGTTTAGTTTAGCCTGCTGC		
Y75	(CTCTT)10	F: TACAATCCACTGTCCACCGC	55	0.375
		R: GGTCTGCATTGGATGTGGGA		
Y78	(ATGTT)14	F: GGCCAAACAGCTACAAGCAC	56	0.757
		R: CGGAAGGTGCGCCTCTATAG		
Y94	(AAGT)7(ATGT)7	F: AGCCAGCGAACATGTACACA	54	0.375
		R: CCGCCTCATTAAATGCTGGC		
Y111	(TC)16	F: CTCTCTCTCTCTCTCTCTCTCTGA	58	0.305
		R: CAAGGGGCTGGACTGCTATG		
Y121	(CTG)18	F: TGCATAGAAACGCTTGCTGA	52	0.347
		R: CGGAAGGATCGTGCAGCA		
Y122	(CTG)19	F: GTTTAGCCTGCTTCTGCTGC	55	0.692
		R: CAAATGCCTTGAGACACCGG		
Y123	(AAT)17	F: GGCAGCACCTGAACACCTAT	55	0.375
		R: AGGGAGCTTGAGGATGGGAT		
Y127	(GCA)10	F: AATGCCTTGAGACACCGGAA	54	0.375
		R: GCTGATTTTCTCCTTGGAAGTCC		
Y133	(GTG)5	F: GGTTGCATTTGTGGTGGTGG	55	0.504
		R: CATGCAGGCCAATTCACGTC		
Y142	(GATA)18	F: CGTAAGGGTGATAACGGAGCA	55	0.555
		R: GGCAGGAACCTGACCACTTT		
Y146	(ATAG)16	F: TGCACTGCGCTGTAACCATA	54	0.590
		R: GGAGAGCTTCTGGGTTGACC		
Y152	(ACATA)10	F: ACCAGCTTTTGACCACCACA	53	0.476
		R: ATCCAGGTCCGTACTTCCGA		
Y163	(AACAT)12	F: CGGGCTGGTACCTCAAATGA	55	0.645
		R: ATCACACCCACCCTCCTACA		
Y187	(AC)6aag(CA)6	F: ATCCCACCCCTAACTCCTCC	57	0.450
		R: GTCCCCCACCTCATAGTCCT		
Y199	(AC)15(<AC>AGAC)(ACAGAC)4	F: ACATGCTGGCTGGCTATACA	53	0.778
		R: TGCAGTGCGTTGCTACACTA		

### 3.4 Genetic Diversity Analysis

Using the 19 SSR loci identified above, we further evaluated the parameters of genetic diversity based on 60 individuals of CGS from six populations. In total, 107 alleles were obtained, with an average of six alleles per locus. Among these loci, both the Y54 and Y127 loci exhibited the lowest polymorphism, with only two alleles detected at each locus, whereas the Y199 locus showed the highest polymorphism with 17 alleles detected. The expected heterozygosity (He) ranged from 0.172 to 0.919, while the observed heterozygosity (Ho) ranged from 0.100 to 1.000. The average values were 0.565 for He and 0.773 for Ho, showing a consistent pattern of Ho being greater than He. Polymorphic information content (PIC) values of all the SSR primers were greater than 0.25, except for the Y152 locus, which was lower than 0.25, indicating moderate to high polymorphism levels (Table 4). Hardy–Weinberg equilibrium (HWE) analysis based on primers revealed significant deviations for most loci, with the exceptions of Y78, Y152, and Y187.

**TABLE 4:** Genetic diversity parameters of the 19 SSR loci.

Locus	Na	Ne	Ho	He	PIC	I	HWE
Y15	4	2.0672	0.967	0.521	0.399	0.7778	***
Y54	2	2	1.000	0.504	0.375	0.6931	***
Y58	3	2.0868	0.957	0.527	0.406	0.7826	***
Y62	4	2.0672	1.000	0.521	0.399	0.7778	***
Y75	3	2.1798	0.950	0.546	0.439	0.8517	***
Y78	9	4.9587	0.733	0.805	0.771	1.7813	ns
Y94	3	2.1352	0.967	0.536	0.421	0.8162	***
Y111	3	2.0219	0.100	0.51	0.386	0.7327	***
Y121	3	1.6202	0.500	0.386	0.321	0.6236	**
Y122	4	3.2301	0.983	0.696	0.633	1.2607	***
Y123	4	2.1706	1.000	0.544	0.433	0.858	***
Y127	2	1.9651	0.867	0.495	0.371	0.6842	***
Y133	4	2.1719	1.000	0.544	0.433	0.8583	***
Y142	7	4.2985	1.000	0.774	0.731	1.6003	*
Y146	14	4.9758	0.450	0.806	0.777	1.963	***
Y152	4	1.205	0.183	0.172	0.162	0.376	ns
Y163	5	3.005	1.000	0.673	0.604	1.2139	***
Y187	12	1.3521	0.217	0.263	0.256	0.7192	ns
Y199	17	11.2034	0.807	0.919	0.904	2.5584	***
Mean	5.632	2.985	0.773	0.565	0.485	1.049	—

*Note:* ns = not significant; *p < 0.05, **p < 0.01, ***p < 0.001.

**FIGURE 4:**
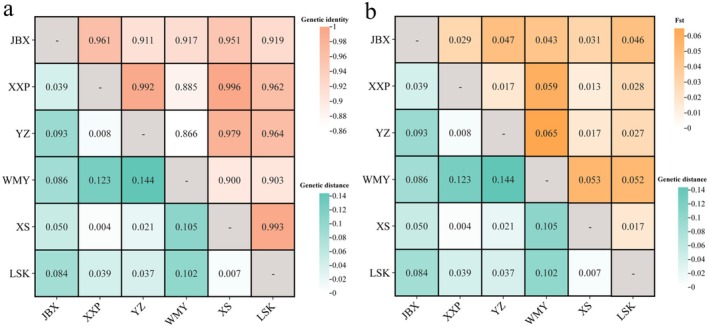
Genetic distance, genetic identity, and Fst of genetic differentiations between each two populations. *Note*: a, genetic distance (lower left) and genetic identity (upper right) values; b, genetic distance (lower left) and genetic differentiation (Fst) (upper right) values.

The average PIC values among these six populations were generally similar. The JBX population had the highest PIC value of 0.490, while the WMY population had the lowest at 0.399. Regarding the average number of alleles, the XS population had the highest (3.789), while the WMY population had the lowest (2.632). The average Ho was greater than the He for all six populations. For different populations, the Ho and He varied, but did not follow a completely consistent pattern: the LSK and YZ populations had the highest and lowest average Ho, respectively, while the JBX and WMY populations had the highest and lowest average He, respectively (Table 5). Nei's genetic distance and genetic identity between any two populations were calculated, generally showing an opposite trend (Figure 4a). The largest genetic distance (0.144) and the lowest genetic identity (0.866) were observed between the YZ and WMY populations. The lowest genetic distance (0.004) and the largest genetic identity (0.996) were observed between the XXP and XS populations. The genetic differentiation coefficient (Fst) showed the same trend as genetic distance (Figure 4b), with the largest Fst observed between the YZ and WMY populations (0.065), and the smallest between the XXP and XS populations (0.013).

**TABLE 5:** Genetic polymorphism parameters of six CGS populations based on 19 SSR loci.

Pop	Na	Ne	Ho	He	PIC	I	HWE
JBX	3.737	2.638	0.770	0.559	0.490	0.998	***
XXP	3.263	2.423	0.783	0.515	0.432	0.876	***
YZ	3.211	2.337	0.715	0.514	0.438	0.875	***
WMY	2.632	2.102	0.764	0.482	0.399	0.770	***
XS	3.789	2.809	0.793	0.543	0.466	0.971	***
LSK	3.526	2.753	0.799	0.536	0.456	0.938	***

*Note:* ***p < 0.001.

### 3.5 Genetic Structure and Cluster Among Populations

**FIGURE 5:**
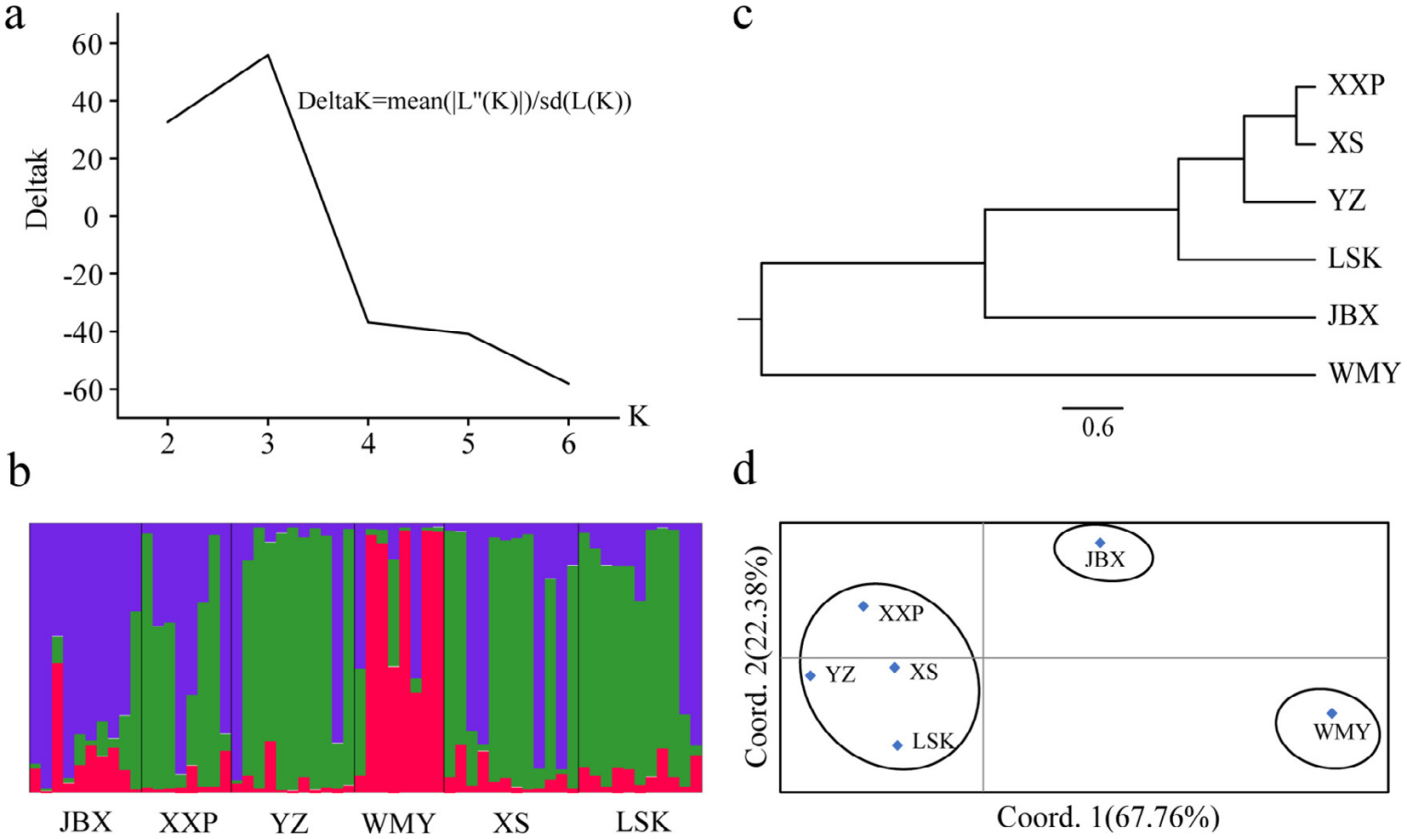
Genetic structure and cluster of six wild CGS populations. *Note:* a, Relation between Δ K and different hypothetical K; b, the population structure of 60 CGS accessions in *K* = 3 clusters; c, UPGMA dendrogram based on Nei's genetic distance; d, PCoA constructed based on genetic distances.

The genetic structure of six CGS populations was analyzed by considering different K hypotheses, based on the change trend of ΔK using Structure software. When *K* = 3, it was observed that the rate of change of ΔK was the highest, and there was a clear inflection point (Figure 5a). Therefore, a genetic structure was reconstructed based on the hypothesis of three CGS subgroups (when *K* = 3). For the two historically natural sites, the genetic structure revealed that the WMY population was predominantly composed of one subgroup (in red), while the YZ population was predominantly composed of another one (in green). For the recently reintroduced sites, the three populations (XXP, XS, and LSK) exhibited genetic structures that were similar to the YZ population. In contrast, the JBX population from the only long‐term reinforcement site exhibited a distinctive genetic structure predominantly characterized by the third subgroup (depicted in purple, Figure 5b).

The six populations were hierarchically clustered using the UPGMA clustering tree reconstruction method (Figure 5c). Based on trichotomic topology, XXP, XS, YZ, and LSK were clustered together, forming the first clade. Then, JBX was divided and formed the second clade. WMY formed the third clade and also the furthest branch. PCoA analysis based on Nei's genetic distances of wild CGS populations showed similar results to genetic structure and cluster analysis (Figure 5d). Three principal components were extracted from the genetic differences of the six wild CGS populations in detail. Among these components, Factor 1 accounted for 67.76% of the variations, Factor 2 accounted for 22.38%, and Factor 3 accounted for 9.87%. Based on the scatter diagram of the first two factors, the six populations can be distinguished into three groups, similar to the cluster analysis.

## 4 Discussion

### 4.1 Development of SSR Markers Based on *de Novo* Genomic Assembly

In this study, we successfully developed genomic SSR markers for the CGS using the NGS and assembly strategies. Despite using only eightfold coverage of the CGS genome for *de novo* assembly, our approach was effective in developing SSRs. Using approximately 400 Gb of raw data, we assembled a total of 15,140,972 scaffolds and identified 316,313 SSR loci based on these scaffolds using the MISA tool. From the initial pool of 200 SSR loci, 19 were selected after rigorous screening, including PCR, polymorphism, and parameter evaluations (Table 3). These 19 SSR loci demonstrated strong performance in assessing the genetic diversity of CGS across 60 wild individuals sampled from six sites. This study presents a significant advancement, especially for species without a representative whole‐genome sequence, such as CGS, where our methodology demonstrates how to address the gap by developing a large number of SSR markers using a shallow genomic assembly strategy, followed by thorough primer screening and validation procedures. Although we only validated 19 of 200 SSR loci in the genetic diversity analysis of this study, our SSR loci list holds considerable potential for further research and marker development in CGS and similar species (Table S1).

The distribution and characteristics of SSRs in the shallow draft genome of CGS were analyzed for the first time in this study. The SSR frequency in CGS was found to be 109.28 loci/Mb, which is lower compared to *Lateolabrax maculatus* (425.06/Mb) (Fan et al. 2021), while the SSR density was 2093.90 bp/Mb, lower than that observed in *Ophiophagus hannah* (9148.21 bp/Mb). Differences in SSR frequency and density across species have been attributed to various factors such as genome complexity, dataset size, SSR mining criteria, bioinformatics tools, and tissue or species types (Wang et al. 2016). Further investigation is required to determine the reasons behind the relatively sparse distribution pattern of SSRs in CGS observed in this study. Analysis of the proportion of various motif repeats in CGS revealed that dinucleotides were the dominant type of SSR repeats (Figure 2), consistent with findings reported in most eukaryotes previously. This pattern has been observed in diverse organisms, such as fungal species in the *Phytophthora* genus (Mathew et al. 2020), plant species such as black pepper *Piper nigrum* (Kumari et al. 2019), and animal species like penguins of the *Pygoscelis* genus (Vianna et al. 2017). However, there are exceptions to this typical characteristic of SSR motifs. Trinucleotides were found to be the most abundant type in *Blattella germanica* (Wang et al. 2015), and tetranucleotides were dominant in *Ophiophagus hannah* (Liu et al. 2019). These variations highlight the diverse nature of SSR motifs across different species and underscore the need for species‐specific analyses.

Among the most abundant dinucleotide repeats in CGS, AC constituted the highest proportion, followed by AG and AT, a pattern consistent with the dinucleotide distribution observed in other species such as the Chinese mitten crab *Eriocheir sinensis* (Xu et al. 2021), Japanese pufferfish *Takifugu rubripes* (Takagi et al. 2003), and *Homo sapiens* (Subramanian et al. 2003). However, this pattern can vary, as AG has been found to have the highest frequency in the horse crab *Portunus trituberculatus* (Song et al. 2008), while AT is dominant in the Chinese shrimp *Fenneropenaeus chinensis* (Gao et al. 2004). Interestingly, we observed that the SSR motif CG was the least abundant dinucleotide repeat in CGS, accounting for only 0.07% of the dinucleotide type. This finding is consistent with observations in many other species, particularly invertebrates, mammals, and plants (Stallings 1992). The scarcity of CG repeats in SSRs across species may be attributed to the special structural effects of repeated CG bases (Edwards et al. 1998).

Interestingly, although the distribution of SSR motifs varied greatly among the five different types, the number of repeat units showed a similar pattern: as the repeat units increased, the number of SSR loci decreased correspondingly (Figure 3). This pattern has also been observed in other species, such as *Pantholops hodgsoni* (Du et al. 2016) and *Ictalurus punctatus* (Tang et al. 2022). This suggests that an increase in repeat units leads to longer motifs, which may affect stability, increase mutation rates, and consequently reduce the number of corresponding repeat types. In summary, the distribution pattern and characteristics of SSRs reflect both long‐term and short‐term evolutionary processes and selection pressures within specific lineages or species. It has been reflected that various taxonomic groups may show similar or distinct patterns of SSR type, abundance, and length preferences (Srivastava et al. 2019).

### 4.2 Genetic Diversity of CGS in the Context of Translocation

Genetic diversity is a crucial aspect of biodiversity assessment, serving as a key indicator of the current status and evolutionary potential of threatened species. In this study, we analyzed the genetic diversity of 60 wild CGS individuals from six populations in the Hunan Zhangjiajie Giant Salamander National Nature Reserve using 19 identified polymorphic SSR loci as molecular markers (Tables 3 & 4). Parameters such as the number of alleles, PIC, and heterozygosity are commonly used to evaluate the genetic level of a species and reflect its gene richness (Botstein et al. 1980). The average number of alleles ranged from 2.632 to 3.789 across the six populations of CGS, indicating a moderate richness of these loci suitable for genetic analysis. Similarly, the average PIC values of the six populations were greater than 0.25 but less than 0.5, suggesting a moderate level of genetic diversity according to the criteria proposed by Botstein et al. (1980). These findings align with earlier studies on the genetic diversity of CGS based on mitochondrial sequences (Tao et al. 2005; Wu et al. 2017). Heterozygosity, which typically reflects the degree of genetic variation within populations (Nei et al. 1975), exhibited an interesting pattern. The average Ho was greater than the average He in all six populations of CGS (Table 4), indicating a potential pattern of heterozygosity excess. Such an excess of heterozygotes is often observed in small populations where only a limited number of individuals participate in reproduction, which may not be representative of populations with a sufficient number of breeding individuals (Balloux 2004). This suggests that the excess heterozygosity observed in CGS populations may indicate a population decline, specifically a shortage of breeding individuals. This underscores the importance of conservation efforts to maintain and restore viable breeding populations of CGS.

Genetic distance, identity, and Fst serve as key indicators for assessing genetic differentiation within and between populations, providing valuable insights into the degree of genetic relatedness. In our study, we observed contrasting trends between genetic distance and identity, while genetic distance and Fst exhibited consistent patterns across different populations of CGS (Figure 4). It was observed that the genetic differentiation between the two historically natural sites, YZ and WMY populations, was the highest, whereas the differentiation between the two recently reintroduced sites, XXP and XS populations, was the lowest. These findings are broadly consistent with our previous results based on mitochondrial gene sequences from the same area (Lan 2023). It is noteworthy that the two historical natural sites, YZ and WMY, are situated in two distinct river drainages, namely, the Lishui and Yuanshui Rivers, respectively. This substantial differentiation suggests a clear separation into two distinct genetic lineages (Liang et al. 2019), or potentially two different species ( *A. davidianus* and *A. sligoi*) according to the newly revised taxonomic system (Chai et al. 2022; Turvey et al. 2019). Such genetic differentiation highlights the importance of considering historical geographic and evolutionary factors in understanding the population structure and evolutionary history of CGS.

The genetic structure analysis revealed the presence of three distinct subgroups (Figure 5a) among the samples collected from the six populations. Consistent with the genetic distances observed, the genetic structure analysis showed that individuals from the two historically natural sites, WMY and YZ, predominantly clustered into separate subgroups (depicted in red and green in the Figure 5b). Intriguingly, individuals from the three populations in recently reintroduced sites, XXP, XS, and LSK, exhibited genetic structures similar to that of the YZ population. However, individuals from the population at the long‐term reinforcement site, JBX, displayed a unique pattern, with their genetic structure primarily dominated by a third subgroup (depicted in purple, Figure 5b). Similarly, the UPGMA clustering analysis and PCoA revealed consistent relationships among the six populations. Three distinct clades (or groups) could be identified: WMY forming the first clade, followed by JBX, and then a third clade comprising LSK, YZ, XS, and XXP (Figure 5c,d). These findings provide further insights into the genetic structure and population dynamics of CGS, highlighting distinct patterns of genetic clustering among populations from different historical and management contexts.

The observed differentiation between the WMY and YZ populations aligns with their distinct historical origins, as they are situated in separate river drainages within the Hunan Zhangjiajie Giant Salamander National Nature Reserve. This divergence likely reflects the natural genetic differentiation of CGS within the reserve. The genetic similarity among individuals from the recently reintroduced populations (XXP, XS, and LSK), all originating from the Lishui River drainage, underscores the general genetic continuity within this lineage. However, the genetic composition of the JBX population, a long‐term reinforcement site, presents a unique scenario. The genetic structure of JBX individuals suggests a mixture of genetic lineages from both the Lishui and Yuanshui River drainages (Figure 5b,c). This suggests the possibility of natural hybridization between the two genetic lineages that originally isolated from two separate river drainages, especially considering JBX's history as the earliest site of CGS translocation in China (Jiang et al. 2022). There were over 1000 individuals reintroduced into this river since 2000 without considering too much of the genetic sources in the past. However, further investigation, particularly with the inclusion of natural family samples and a broader panel of highly polymorphic SSR markers, will be necessary to validate this hypothesis. Nonetheless, JBX serves as a crucial site for understanding the complex genetic interactions and conservation implications of translocated CGS populations, and it presents an intriguing opportunity to study the genetic dynamics and conservation genetics of wild CGS under the context of translocation.

## Author Contributions

Wansheng Jiang (the first corresponding author) conceived the study; Mingyao Zhang, Qiang Zhou, Yixing Xie, Cheng Wang, and Fengjiao Wu collected the data; Mingyao Zhang and Yixing Xie analyzed the data and wrote the first draft; Wansheng Jiang and Ying Wei revised the first draft; Zhiyong Deng and Zhiqiang Liang improved the draft; all authors read and approved the final manuscript.

## Ethics Statement

The collection and sampling of the specimens were reviewed and approved by the Ethics Committee of Jishou University (No: JSDX‐2020‐0019), according to the “3R principle” (Reduction, Replacement, and Refinement) that is required by the National Ministry of Science and Technology (No. 398 [2006]).

## Conflicts of Interest

The authors declare no conflicts of interest.

## Supporting information


**Table S1.** The SSRs of Chinese giant salamanders identified from the draft genome in this study.

## Data Availability

All data generated and analyzed during this study are included in this published article and the Supplementary Table S1.
